# Contrary to Myth, Older Adults Multitask With Media and Technologies, But Studying Their Multitasking Behaviors Can Be Challenging

**DOI:** 10.1093/geroni/igz029

**Published:** 2019-10-16

**Authors:** Anastasia Kononova, Pradnya Joshi, Shelia Cotten

**Affiliations:** 1 Department of Advertising + Public Relations, College of Communication Arts and Sciences, Michigan State University, East Lansing; 2 College of Business, West Texas A&M University, Canyon; 3 Department of Media and Information, College of Communication Arts and Sciences, Michigan State University, East Lansing

**Keywords:** Media-use research methods, Multitasking with media and ICTs, Older adults

## Abstract

**Background and Objectives:**

The study’s objective was to explore older adults’ (aged 65 or older) descriptions of behavior related to multitasking with traditional and newer media/information and communication technologies (ICTs) and perceived benefits of such behavior, along with older adults’ preference for research methods used to study their multitasking behaviors. Employing common media-use measures that heavily rely on self-reporting in populations of older adults is challenging, especially given that patterns of media/ICT use are becoming increasingly complex. Cumulatively, people spend more time using media than they are aware of because of the tendency to use some forms of media simultaneously. As cognitive ability deteriorates with age, self-reported recollection of complex patterns of media/ICT use, such as multitasking, among older adults increases the threat to data accuracy.

Research Design and Methods:  Twenty-eight community-dwelling older adults in a Midwestern U.S. state participated in in-depth interviews (average length was 40 minutes) to discuss their use of traditional and newer media/technologies in combination with other activities and outline methods researchers should use to study such behaviors.

**Results:**

Participants reported they engaged in multitasking behaviors similar to those of younger generations, with the difference in the higher extent of using traditional media and ICTs. They talked about multitasking with radio and television for “background noise” as being a rewarding experience. They perceived the effects of multitasking to be detrimental to attention and performance and attributed this media-use habit to individual psychological and demographic differences. They preferred ethnographic observation and keeping a paper-and-pencil diary as research methods to study multitasking among their peers. Data-logging methods were less popular because they raised privacy concerns among interviewees.

**Discussion and Implications:**

Different types of traditional and newer media and technologies could be used differently in situations that require older adults to focus, relax, or be efficient. The findings suggest that future researchers strive for a compromise between data access and data accuracy when they select a research method to study media use among older adults.

**Translational Significance:**

Older adults, who have been multitasking with traditional media (print, radio, television) for decades, are incorporating newer forms of ICTs (smartphones, computers, e-readers) as part of these behaviors; and, despite understanding that multitasking creates distractions and increases the chances of making errors or missing information, they associate media/ICT multitasking with task enjoyment, task focus and productivity, loneliness management, and keeping up with the news. Study participants are less ready to participate in data-logging media multitasking research and preferred keeping a time diary and participant observation.

The world of media and information and communication technologies (ICTs) is constantly evolving. Falling behind on updating technology-use skills can be detrimental to one’s personal, professional, and social life, especially among older adults (aged 65 and older), who generally exhibit slower rates of technology adoption (Cotten, Yost, Berkowsky, Winstead, & Anderson, 2017; Green & McAdams, 2003; Selwyn, Gorard, Furlong, & Madden, 2003; Selwyn, 2004; Teo, 2001; Vroman, Arthanat, & Lysack, 2015). Older adults face numerous barriers to adjusting to social changes dictated by technological progress (Berkowsky, Sharit, & Czaja, 2018). However, the number of older adults who own or use new ICT devices (such as smartphones, tablet and laptop computers, wearable devices, and game consoles) is increasing, reflecting the overall growth in consumer availability of these devices (Anderson & Perrin, 2017). In this study, we consider media and ICTs to encompass a wide range of tools through which older adults receive information and communicate with others, including (1) mass media (print, radio, television), defined as channels that transmit a message from a single or few communicators (e.g., a television production company) to a large number of recipients and (2) technologies that allow communication and information transmittal in interpersonal and mass settings (e.g., the Internet; smartphones, cellphones, and landline phones; desktop, laptop, and tablet computers; e-readers, etc.).

New media and ICT devices are portable, allowing them to be used in various locales (e.g., work, home, transit) and social settings (e.g., alone, with friends, with strangers; Schrock, 2015; Zhang & Zhang, 2012). Devices such as smartphones promise to offer greater efficiency in completing everyday tasks due to the convergence of multiple functions within one screen (e.g., Deng et al., 2019); yet they also frequently distract users from one task to attend to another task (e.g., Levine, Waite, & Bowman, 2007).

The emergence of new media and ICTs has led individuals to develop new habits, such as multitasking (defined in general as performing several tasks within the same time frame). Over the past three decades, scholars have investigated multitasking when at least one of the tasks is media/ICT-related. The tasks can be simultaneous, implying concurrent exposure and/or actions (e.g., driving and listening to radio), or they can require frequent motor and/or attentional switching (e.g., checking a phone while reading a book) (Salvucci & Taatgen, 2010). Notably, media- and ICT-related multitasking is particularly widespread in societies with high media/ICT availability and ownership (Jeong & Fishbein, 2007; Kononova, 2013; Kononova, Zasorina, Diveeva, Kokoeva, & Chelokyan, 2014). These societies, typically defined as “industrialized,” “modern,” and “economically developed,” also have higher percentages of older adults in their populations as well as higher aging rates (Wagner, Hassanein, & Head, 2010; World Bank, 2017; United Nations, 2017).

Although media/ICT multitasking behaviors have been widely studied in populations of young adults and children (e.g., Foehr, 2006; Kononova, 2013; Kononova et al., 2014; Rideout, Foehr, & Roberts, 2010), few scholars have analyzed media multitasking habits across multiple age groups and generations (e.g., Carrier, Cheever, Rosen, Benitez, & Chang, 2009; Voorveld & van der Goot, 2013) and none—to our knowledge—has focused specifically on adults aged 65 and older. We know little about how multitasking with media/ICTs, which often increases cognitive demands, is integrated in daily routines of older adults whose cognitive ability starts to deteriorate (e.g., Buckner, 2004). Moreover, media researchers face the challenge of finding appropriate methods to study complex patterns of media/ICT use in older age groups. Methods based on self-reporting may not be suitable in such populations due to higher chances of cognitive bias and increasingly fragmented media use that is difficult to keep track of and recall in a questionnaire (Southwell & Langteau, 2008). Thus, it is important to determine appropriate methods of studying media-use patterns in this population. Such methods must not only produce data of good quality but also be acceptable by older research participants.

In this study, we examined older adults’ accounts of multitasking with media and ICTs to determine the characteristic patterns and perceived effects of this behavior in this population. Study participants shared their perceptions of research methods to measure media-use behaviors, indicating the ones they would be most comfortable with. Our results shed light on the role of traditional and new media in lives of our older adult participants (most of whom were members of socioeconomic and cultural majorities) that can be used to inform development and implementation of future research strategies to investigate reducing negative effects of age-related changes. The present study also provides a basis for developing methodological guidelines to measure complex media-use behaviors in the population of older adults in the United States.

## Literature Review

### Benefits for Older Adults of Using and Multitasking With Media and ICTs

Media and ICT use is associated with a great number of emotional and cognitive benefits, both observed and perceived, including increased satisfaction of communication, social interaction, information, and entertainment needs; greater psychological well-being; higher self-reported quality of life; and higher life efficiency among older adults (Berkowsky et al., 2018; Furlong, 1989; Heo, Chun, Lee, Lee, & Kim, 2015; Hill, Betts, & Gardner, 2015; Irizarry & Downing, 1997). Increased interaction with friends and family (specifically, grandchildren) via technology helps older adults cope with limited mobility and distress of losing loved ones (Antonucci, Ajrouch, & Manalel, 2017; Francis, Kadylak, Makki, Rikard, & Cotten, 2018; Opalinski, 2001; Weatherall, 2000). Older adults’ use of media and ICTs has been negatively associated with aging-related loneliness, depression, and boredom (Carpenter & Buday, 2007; Chen & Persson, 2002; Chopik, 2016; Cotten, Anderson, & McCullough, 2013; Cotten, Ford, Ford, & Hale, 2012, 2014; Czaja, Boot, Charness, Rogers, & Sharit, 2017; White et al., 2002). Using radio and television while doing something else (i.e., multitasking) has been described as an enjoyable experience associated with older Finnish adults’ well-being (Niemelä, Huotari, & Kortelainen, 2012).

Media/ICT use has been shown to provide cognitive stimulation to older adults. Engagement in cognitive and leisure activities that involve traditional media use (print, radio, television) is negatively associated with cognitive decline and developing dementia, specifically, Alzheimer’s disease (Crowe, Andel, Pedersen, Johansson, & Gatz, 2003; Fabrigoule et al., 1995; Friedland et al., 2001; Fritsch, Smyth, Debanne, Petot, & Friedland, 2005; Kondo, Niino, & Shido, 1994; Scarmeas, Levy, Tang, Manly, & Stern, 2001; Verghese et al., 2003; Wilson et al., 2002). In the past decade, media-use research on aging has shifted to studying the usage of the newest ICTs and its potential correlation with cognition. The frequency of computer and Internet/E-mail use, for example, is a significant predictor of lower risk of dementia and slower cognitive decline (Almeida et al., 2012; Xavier et al., 2014), with computer gaming being viewed as a particularly beneficial and enjoyable activity for older adults (Boot et al., 2018). Additionally, the Internet has been used to implement online cognitive training programs for those who are 65+ years old and has shown to improve healthy older adults’ cognitive functioning (Klimova, 2016). However, limited research is available on the effects of media/ICT multitasking on cognitive function in older individuals. One study, Neuroracer, implemented two simultaneous tasks within a driving simulation video game in which older individuals had to control a car on the road while reacting to additional stimuli (shapes of different colors). Playing this multitasking video game over time improved older players’ executive function (Anguera et al., 2013). Nonetheless, little evidence is available with regard to, first, cognitive effects of multitasking with multiple media and devices among older adults and, second, the media multitasking habit rather than effortful videogame playing.

### Media and ICT Use and Multitasking Among Older U.S. Adults

#### New media/ICT use

Older U.S. adults are increasingly using new media technologies, such as the Internet, smartphones, tablet computers, and social media (Anderson & Perrin, 2017). Sixty-seven percent of older adults used the Internet in 2016, compared with only 12% in 2000. Smartphone ownership increased from 11% to 42% from 2011 to 2016. The number of older adults who have a mobile phone increased from 69% in 2012 (Smith, 2014) to 80% in 2016, although the smartphone ownership rate is lower. Tablet computer ownership grew from 1% in 2010 to 32% in 2016; the use of social media climbed from 2% in 2008 to 34% in 2016. One in five adults aged 65+ (19%) had an e-reader in 2016 (Anderson & Perrin, 2017).

#### Traditional media/ICT use

Although the rates of new media/ICT use are increasing among older adults, they are relatively low compared with those for all U.S. adults (Anderson & Perrin, 2017). Despite the slower pace of new media and ICT adoption, older adults are heavy users of traditional media. This age group constitutes an audience that is the most loyal to print outlets—on average, they are more likely to read newspapers and magazines than are other members of the U.S. population (Simmons Research, 2016). This age group has the highest readership rates of daily and Sunday newspapers, despite the general decline in the readership of these publications. Over one-half of older U.S. adults read newspapers regularly in 2014 (Mitchell & Page, 2014).

Older adults are heavy users of television. More than 85% of U.S. adults aged 65+ watched television on an average weekday and weekend in 2016, which indicated a higher viewership compared with overall rates for the U.S. population (Simmons Research, 2016). Two in three older adults (67%) had a cable television subscription in 2016, and one in three (38%) had a satellite dish in the household. Cable and satellite subscription rates of older adults were higher than the rates were for the overall U.S. population in 2016.

Radio listenership for older adults was lower than for the average adult U.S. population in 2016, with 61% listening to radio in general and 48% doing so while driving. Fifteen percent of those aged 65+ were subscribed to Sirius satellite radio in 2016, which was higher than the percentage for the U.S. population as a whole (Simmons Research, 2016).

#### Media/ICT multitasking

Earlier researchers (e.g., Carrier et al., 2009) found that older generations of media users engaged in media multitasking to a lesser degree than their younger counterparts. Yet those aged 50–65 have been found to multitask with media as much as younger individuals: Voorveld and van der Goot (2013) found that Dutch adults who were 50–65 years old expressed preference for combining new and traditional media, such as checking E-mail while browsing websites, listening to radio, watching television, and reading newspapers. Finnish adults who aged 60 and older listened to radio and kept television on while doing something else, which they evaluated being as a pleasant experience (Niemelä et al., 2012). According to data from Simmons Research (2016), U.S. adults aged 65+ reported doing the following activities very often, often, or somewhat often: reading magazines or comics while watching television (91.5%), pairing television and radio use (36.71%), browsing websites while watching television (32.17%), checking E-mail on a computer with television on (37%), using a computer for instant messaging during television watching (21.82%), playing video games with television on (33.65%), and watching television while using a cell phone to talk (30.87%), text (16.83%), or visit websites (11.52%).

### Why Study Media/ICT Multitasking in Older Adults?

Media use is often combined with other activities. In studies that measure frequency of media use, accounting for, as well as distinguishing between, sole-media use and media multitasking provides greater accuracy. This distinction is important because different levels of frequency of sole- and shared-media use may be associated with different levels of cognitive stimulation. Evidence derived from studies with younger adults, while inconclusive (Wiradhany & Nieuwenstein, 2017), suggests a negative correlation between habitual media multitasking and executive control function (Ophir, Nass, & Wagner, 2009). Ophir and colleagues (2009) correlated self-reported media multitasking with the results of executive function assessment in college students and found that those who reported higher levels of multitasking with media, traditional and new, performed more poorly on task switching and distraction-filtering cognitive tests. The explanation of this relationship, Ophir and colleagues (2009) suggested, could be correlational rather than causational, with college students who had poor cognitive ability to filter distracters being “breadth-biased,” that is, attending to multiple sources in the environment, thus being more prone to multitasking. Furthermore, cross-sectional experimental studies established that when younger adults multitask, they perform poorly on media and non-media tasks (e.g., poor memory for and comprehension of media messages; weaker counterarguing; Armstrong & Chung, 2000; Jeong & Hwang, 2012; Kononova, Joo, & Yuan, 2016; Srivastava, 2013). Survey research links media multitasking in young adults with self-reported negative outcomes, such as higher distractibility, increased time costs, and decreased chances of finishing work (Bowman, Levine, Waite, & Gendron, 2010; Junco & Cotten, 2011; Levine et al., 2007). Despite cognitive costs, people describe media multitasking as being an enjoyable experience (Chinchanachokchai, Duff, & Sar, 2015; Jeong & Hwang, 2016). A comprehensive qualitative study of perceived benefits and costs of multitasking among undergraduate students has shown similar findings: While young adults perceive multitasking to be distracting, addicting, creating chaos, and bringing disengagement, they also enjoyed it, engaged with it, habituated to it, and associated it with control and efficiency (Bardhi, Rohm, & Sultan, 2010).

Although we know that older adults multitask with media and slowly adopt new ICTs, there is a dearth of evidence to understand the effects of this behavior on their cognitive and emotional health. Does constant distraction created by media/ICTs negatively affect cognition on a long-term basis? How effective is purposeful, focused multitasking within one medium (Anguera et al., 2013)? Does multitasking improve psychological well-being? Although this study does not answer these questions, it prepares the ground for future studies to do so. We start by focusing on how, why, and with what perceived effects older adults multitask with media/ICTs and what methods they suggest researchers use to study this phenomenon.

In summary, older U.S. adults have been shown to be heavier users of traditional media and slower adopters of new media and ICTs. Given that only fragmented data from academic research and industry are available with regard to these adults’ media- and ICT-related multitasking behaviors, first, we asked:

**RQ1**: What are the patterns of multitasking with traditional and new media and ICTs among in-depth interview participants who are U.S. adults aged 65 and older?

Our qualitative investigation of media and ICT multitasking among older adults went beyond identifying the patterns as we strived to understand additional meanings that older adults associate with this behavior.

**RQ2**: What are the associations and meanings that older adults ascribe to the behavior of media and ICT multitasking?

### Measuring Media Use and Multitasking

Applied and academic research in the field of media and ICTs is often based on survey and diary self-report measures of media use for which participants estimate frequency of media/device use, time spent with a medium/program/device, and attention paid to mediated content, among other variables (Fishbein & Hornik, 2008; Southwell & Langteau, 2008). Most commonly used measures of media multitasking are based on respondents’ recollection of time shared between several media/ICT-related tasks (e.g., Ophir et al., 2009). Empirical evidence confirms that recall error increases with age, reducing the quality of self-reported data (Southwell & Langteau, 2008). There is a dearth of studies that focus on older adults’ concurrent uses of media and ICTs and a potential relationship between aging and accuracy of self-reported media- and ICT-related multitasking data.

Over the past three decades, developments in tracking technologies have yielded less-biased methods of data collection rooted in data logging, ranging from Nielsen’s “peoplemeters” that track basic television viewing patterns (Southwell & Langteau, 2008; Taneja & Mamoria, 2012) to mobile applications that record the time and nature of smartphone usage (e.g., Deng et al., 2019). In recent years, communication and media practitioners have moved from capturing mono-use of specific media and devices to measuring multidevice use (Taneja & Mamoria, 2012) to account for multitasking behaviors. For example, Facebook tracks how the social network’s users access it on multiple devices and provides cross-platform services to advertisers (Facebook Blueprint, n.d.). Academic research has explored patterns of switching between mobile apps (Deng et al., 2019) and computer programs (Yeykelis, Cummings, & Reeves, 2014) within the same screen device. To our knowledge, no studies have applied data-logging methods to explore media- and ICT-related multitasking among older adults.

App-tracking and screen-capturing software is available to researchers at low cost, yet its systematic use is still in the infancy phase (e.g., Vanderwater & Lee, 2009). It is also important to note that data-logging information is mostly available through new media devices, such as smartphones, tablets, laptops, and desktop computers. Older adults have lower rates of using these technologies (Anderson & Perrin, 2017) and are also skeptical about sharing personal data. Studies by multiple researchers show that older adults are concerned about their privacy when using social media, computers, E-mail, and the Internet (e.g., Carpenter & Buday, 2007; Gatto & Tak, 2008; Jiang et al., 2016; Leist, 2013; Xie, Watkins, Golbeck, & Huang, 2012). Thus, we asked our participants about their perceptions of acceptable methods to use to study complex media-use patterns.

**RQ3:** What methods would our in-depth interview participants who are U.S. adults aged 65 and older prefer for researchers to use to capture their complex media- and ICT-related multitasking behaviors?

## Method

### Recruitment and Sample

We conducted semi-structured, face-to-face interviews with 28 older adults who were 65 years of age or older (see Table 1 for demographic characteristics). We focused on older Baby Boomers and members of the Silent Generation in the United States who were born between 1925 and 1950 (Strauss & Howe, 1991). The participants were recruited through a local senior center in a metropolitan area in a Midwestern U.S. state and through a university-based online recruitment system that allows access to a pool of local community participants (*N* > 3,500; 9% are adults older than 51). Convenience sampling strategies were used: we distributed flyers at the senior center and sent an electronic announcement through the online recruitment system. Participant selection criterion (being 65 years of age or older) was clearly communicated in the flyer and announcement. Potential participants were asked to contact researchers by phone to learn more about the study and, if they agreed to participate, schedule the time of an interview. The interviews were conducted at locations convenient to the participants, including participants’ homes, the senior center, and the university campus. The recruitment was based on voluntary participation. All participants who contacted the researchers were willing to participate in the study, but two had to cancel interview appointments due to changes in their schedules. Initially, we planned to interview 30 older adults, but because data collection reached the point of saturation, with participants providing similar insights on the topic, the decision was made to stop recruiting and interviewing.

**Table 1.** Demographic Characteristics of Study Participants

Demographic characteristics	Descriptive information
Age	
Mean	74.11
Median	73
Standard deviation	7.41
Range	65–90
Gender	
Females	18 (64%)
Males	10 (36%)
Race	
White/Caucasian	100%
Marital status	
Married	11 (39%)
Separated/divorced	4 (14%)
Single	6 (21%)
Widowed	5 (18%)
Other	2 (8%)
Education	
Some high school	1 (4%)
High school	4 (14%)
Associate degree	3 (11%)
Bachelor’s degree	13 (46%)
Graduate degree	7 (25%)
Occupation	
Retired	22 (79%)
Prior/current occupation	
Administrative/managerial	8 (28%)
Clerical/white collar	2 (8%)
Craftsman/blue collar	1 (3.5%)
Educator/researcher	5 (18%)
Homemaker	1 (3.5%)
Professional/technical	4 (14%)
Sales/service	4 (14%)
More than one	3 (11%)
Annual income	
Not reported	6 (21%)
$10,081–$14,100	3 (11%)
$14,101–$18,000	2 (7%)
$22,201–$26,160	2 (7%)
$26,161–$30,000	1 (3.5%)
$30,001–$48,000	1 (3.5%)
$48,001–$75,000	7 (25%)
$75,0001–$99,600	5 (18%)
More than $100,000	1 (3.5%)
Religion	
Not reported	8 (28%)
Christian	13 (46%)
Catholic	5 (18%)
Lutheran	2 (7%)
Mormon	1 (3.5%)
Presbyterian	1 (3.5%)
Protestant	1 (3.5%)
United Methodist	1 (3.5%)
Christian (no type specified)	2 (7%)
Jewish	3 (11%)
Other	1 (4%)
Not religious	3 (11%)

### Materials and Procedure

All materials and study procedures were approved by the Institutional Review Board at a large Midwestern U.S. university, where we conducted this study. Each participant was given time to read the consent form. The option of not participating was provided. After participants signed the consent form, we began the interview. Participants could withdraw from the interview at any time and choose to not answer any question. No participants refused to answer questions or stopped participation. Each interviewee received $20 in compensation for participation.

Each interview lasted, on average, for 40 min. Each participant was asked a predetermined set of open-ended questions about their use of traditional media and new media, and the ways in which they multitask with media. Traditional and new media categories were clearly defined by listing types of media and devices each category comprised. Examples of traditional media/technologies included newspapers, magazines, books, radio, television, and landline phones. Examples of new media included Internet-enabled, computer-based, and mobile devices, such as desktop, laptop, and tablet computers; e-readers; mobile phones (both smartphones and cell phones only capable of handling voice calls and text messages); and video games. The question about media multitasking started with the following definition: “It often happens that we use media while doing something else. We call this behavior media multitasking….” To avoid confusion, we used the general term “media” to refer to both mass media, such as television, as well as ICT devices, such as smartphones and tablet computers. We asked participants whether our definitions were clear throughout the interview to ensure understanding. At the end of the procedure, participants shared their ideas about innovative methods that researchers could use in the future to measure media use and media multitasking in their age group. They were probed to share their thoughts about participant and digital camera observation, keeping a diary, and having tracking (data-logging) software installed on electronic devices. The interviews combined observation and informal information sharing, and participants were encouraged to share their opinions and thoughts.

We recorded the interviews with the permission of participants. Data anonymity and confidentiality were guaranteed. An online service was used to transcribe de-identified interview audio files. We used an inductive approach to analyze the transcripts and performed exploratory thematic analysis to identify common patterns and ideas that were later combined into several themes.

Two researchers iteratively analyzed the transcripts, meeting regularly to ensure consistency in their coding of the transcribed materials. Transcript coding led to the identification of 51 patterns and ideas that were combined into larger categories (codes) after extensive discussion. The codes served as the basis to form themes. We selected multiple quotes corresponding to each pattern and idea. We then aggregated the quotes for each code. The quotes most representative of each theme were picked per agreement of all researchers.

## Results

### Media and ICT Use

Participants’ most common traditional media- and ICT-use behaviors included watching television (*N* = 28); listening to radio at home and while driving (*N* = 24); and reading newspapers (*N* = 23), magazines (*N* = 21), and books (*N* = 18). Most commonly used new media and ICTs were cell phones (*N* = 19), desktop computers (*N* = 18), or laptop computers (*N* = 15). Ten participants used tablet computers, and seven had smartphones. Participants used new media devices to play preinstalled games (e.g., solitaire on a computer), shop and search online, and use social media. Fifteen participants used at least one social media platform, Facebook being the most popular one. The most popular media multitasking pairs included television–print media, television–computer, television–cell phone, and computer–cell phone.

### Preferred Methods to Measure Complex Media/ICT Use

Eighteen participants agreed to “host” a researcher for several hours a day in the course of a week (participant observation), and 18 participants suggested that filling out a paper-and-pencil diary two to five times a day in a week would be a convenient way to measure their media use. Only one-quarter of participants agreed to answer diary questions by phone texting (*N* = 7) and phone calling (*N* = 7), and five suggested they would do it by E-mail. Half of the interviewees (*N* = 14) agreed on more precise but more intrusive media-use tracking methods, such as cameras installed in participants’ homes (mostly living rooms and kitchens) and software installed on electronic devices, mostly desktop computers.

### Media and ICT Multitasking: Themes

#### Background noise

Participants said they used traditional electronic media more heavily than new media/ICTs, but they often combined media use with other activities. Radio and television were the most commonly used media, and music was the most preferred media content in multitasking situations. Participants often mentioned using radio and television for “background noise.” They kept them on while eating, doing house chores, crafting, fixing things around the house, and driving (e.g., radio in the car). Some participants referred to television as a medium to listen to rather than to watch. Having radio or television in the background was perceived to help complete primary tasks efficiently and “save time.” Music, for example, could make it feel “easier to maintain the pace required” (Participant 10, male, 68). On one hand, multitasking was driven by procrastination and the perception that dull activities became easier, quicker, and more pleasant when paired with a media task.

I’m doing some needlepoint project which I find, frankly, boring but I promised somebody I’d do it for them. And so, I put the TV on as a background noise for that. Participant 21 (female, 73)

On the other hand, some interviewees engaged in multitasking because they wanted to prolong the enjoyment with their favorite habits, that is, double the pleasure from simultaneous tasks. For instance, the love of music motivated several participants to listen to CDs and radio in their cars and even acquire new media devices and services, such as iPods and satellite radio, to make music available at any time and location.

When activities were perceived as physically and cognitively exhausting, using media and ICTs helped participants “stay on track.” First, they would turn on a specific type of music to play simultaneously with a demanding task for “a calming effect” (Participant 13, male, 71). Second, they would take a break for an entertaining media activity (e.g., playing a computer game of solitaire) to “clear mind” and “sort of relax” (Participant 27, male, 77).

Additional reasons for keeping media on were avoiding silence or undesirable noise, escaping boredom, and managing loneliness.

A lot of times in one day I’ll be sewing and cooking and watching TV, intermittently back and forth or on the computer. […] It’s a matter of trying to keep busy and not go stir crazy for me…. Well one of the reasons I do here is because living alone is maddening when there’s no other voices and I have the TV on for my basic company. It’s my friend. Participant 6 (female, 73)

#### Relevant versus irrelevant

Multitasking with traditional electronic media provided a connection with the world. Television and radio were kept on as if participants feared to miss important information. Many participants admitted that although they kept television and radio on for most of the day, they did not watch or listen to these media and got busy with other things instead. Only when something “relevant” caught their attention did they stop or reduce other activities to a minimum to focus on electronic media content.

I’ll make breakfast, I’m reading the newspaper, I’ve got the TV on in the other room to the news loud enough that if I hear something that tweaks my interest I can pop around the edge of the fireplace and take a look from the kitchen to see what the issue is that they’re bringing up, so I operate very much that way. Participant 13 (male, 71)

At the same time, participants said they were overwhelmed by the amount of irrelevant “boring” and “annoying” content. Thus, multitasking with media and ICTs was driven not only by the need to monitor important and relevant information but also by the need to avoid worthless information. Sources of such information varied from radio and television to overly talkative friends and family on the phone. Interviewees said they turned to print media and books when they wanted to avoid advertisements and other irrelevant content from electronic media.

‘Cause frankly I find most TV shows pretty boring. Participant 21 (female, 73)

When the ads come on, I pick up a book…. I do not like most of the ads on television. Participant 22 (male, 80)

Avoiding undesirable content in traditional media motivated participants to use new ICTs (playing computer games, browsing the Internet, and checking E-mail and social media).

#### Reading requires focus

Participants did not extensively multitask with print media. Using newspapers was associated with pleasant relaxation and time allocated for oneself. Participants mentioned relaxing and slow-pace activities that did not interfere with the joy of reading, such as taking a bath, drinking coffee in the morning, or listening to music. Furthermore, participants indicated a clear preference for print media over any type of new digital equivalents. They preferred having a paper product in hand over the efficiency of electronic information processing.

I love reading in the Nook, but I think I prefer a book, the printed book. I love to have a book in hand with print. ...I don’t know. There’s just something about that. Participant 11 (female 69)

#### Perceived cognitive price of multitasking

Several interviewees said they tried to avoid multitasking with media and ICTs, and a few denied engaging in it. This behavior, for example, contradicted one participant’s beliefs about mindfulness and positive effects of focusing on one task (Participant 2, male, 66). Media/ICTs, in this participant’s opinion, created constant “unhealthy” distraction. Distraction entailed cognitive costs and negative consequences. Among the most commonly mentioned consequences was the lack of concentration on one activity that led to potentially poor task performance, making errors, and missing information.

If I’m watching [TV] while I’m doing crocheting, then I can’t concentrate on the program that’s going on the TV and then I miss a lot of it. Participant 24 (female, 77)

Another negative outcome was a danger to one’s life—whether it was related to losing control over one’s own body or suffering consequences from someone else’s multitasking.

If you multitask, you’re much less effective… […] Yeah, you’re just not as accurate…. Especially, if I’m trying to do too many things at once. It’s easier to make a mistake, it’s easier to trip and fall. Participant 6 (female, 73)

When I see policemen multitasking turning left-hand corners, on the phone, going through almost red lights, I’m not happy, folks. Participant 26 (female, 76)

#### Who are multitaskers? Personality, gender, age

Participants suggested that differences in media- and ICT-related multitasking behavior and the perceived effects occurred not only because of the types of tasks individuals complete and media structural features but also because of personal characteristics of multitaskers. Some interviewees focused on their individual psychological characteristics to explain why they multitask with media/ICTs. A few of them attributed the inability to “sit still” to “short attention span.” One participant suggested that the decision to multitask comes automatically, without thinking, through “a sixth sense” (Participant 28, female, 65).

I don’t like to waste time. […] I cannot stand to be doing nothing creative or purposeful, so I do it. […] I can sit there and cut out skeletons [for crafts] and different things, or fix up a gift for my granddaughter while I’m [watching television].... Participant 14 (female, 73)

Interview statements of predominantly male participants revealed that gender was another perceived personal characteristic connected to media use and multitasking. Female spouses were often described as technologically savvy (i.e., having a smartphone or tablet). Male interviewees claimed that their female spouses were heavier multitaskers than they were. While they tended to be skeptical of copying their wives’ multitasking habits, they expressed eagerness to accept help from their partners to learn about new ICTs.

A few participants “blamed” difficulties in multitasking on their age. They referred to their “before-the-retirement” experiences at work where they said they used to multitask much more frequently. When productivity was a required outcome and things had to be done, multitasking helped. For some, multitasking was perceived to be necessary to focus or make a job more exciting, and for others, it was associated with job-related stress. Discussing age, participants compared beliefs about media and ICT use “now and then” as well as expressed little understanding of younger generations perceived to be digital media and multitasking natives.

I remember as a child, my father telling us that we couldn’t listen to the radio when we were in the car because it was too big of a distraction. So that shows you how things have changed. Participant 10 (male, 68)

I don’t get it. And then all these kids, walking around, they don’t even hear trucks or cars or anything. They walk out in front of them. Participant 18 (male, 69)

### Measuring Complex Media Use: Themes

#### Seeing the value

Participants expressed skepticism toward digital camera and participant observation methods to measure media/ICT use and multitasking, which was mostly related to the issue of data “objectivity.” They suggested that awareness of a camera (in the case of digital observation) or a researcher’s presence in the home would alter the behaviors of residents and require time to forget about the fact of being watched.

So I can’t make breakfast in my underwear anymore? Participant 2 (male, 65)

I don’t think it would be real because […] the person would be performing…. Participant 4 (male, 85)

I feel I have been spied upon…. Participant 5 (female, 86)

Another overwhelmingly popular response to the question about observation methods was related to participants giving little value to their life routines. They often described their everyday existence as “not interesting” and “boring” and concluded that observation data would be of little value to researchers.

What about if we wanted to come in your home and observe you, like I mentioned, somebody coming in for a few hours a day for several days? Interviewer

I might have to pay you for that. Participant 1 (female, 65)

You’d sit there and watch me watch television most of the time. There wouldn’t necessarily be anything to see […] I’d be sitting there saying “Are you bored? What are you really getting out of this?” Participant 16 (male, 72)

Other thoughts about observation methods dealt with concerns related to space limitations and time commitments. For example, cameras couldn’t be installed all over the house, and researchers would have to arrange certain times for observation to ensure participants were not busy. The limitations of observational methods could be overcome, according to participants, by installing “far more reliable” data-logging software on personal devices to track usage (Participant 7, female, 67) or by asking participants to self-report their media- and ICT-use behaviors.

#### Privacy is an issue

Although data-logging methods could provide precise information about media and technology use, an overwhelming number of interviewees called this method, along with digital camera observation, invasive. Participants used terms like “intrusion,” “Big Brother,” “reality TV,” “spying,” and “threat to security” to describe their privacy concerns. Being private, being used to living alone, and feeling uncomfortable were listed as reasons for not allowing data collection via camera and tracking software.

Home is home. That’s where it stays. Participant 20 (male, 81)

People can hack your things. You know? […] I think part of it is growing up in New York. I’m the kind of person who locks their car everywhere. My door is always locked. Participant 23 (female, 73)

Participants who were worried about privacy preferred to use a time diary method, which they described to be the least invasive. A few said they would agree to digital observation and data-logging software installation if they could view the footage before researchers do and turn off the “surveillance” at any time.

#### Paying extra for invasion

There was an agreement that participation in studies that use invasive methods of data collection should earn a higher compensation compared with that for less invasive studies. Two participants pointed out that they would agree to participate in an “intrusive” study if they were interested in it and clearly understood its goal.

#### Other recommendations

Participants perceived in-person observation and keeping a paper-and-pencil diary as “comfortable” methods of data collection. They suggested taking into consideration a number of factors. Agreement to a participant observation study was not a unilateral decision and required consulting other members of the household, mostly partners and children. In addition, it required a time commitment because participants, especially those who were retired, had busy schedules.

One thing I’m finding is seniors are never home. They’re out and about. Participant 15 (female, 66)

Keeping a time diary was considered a good solution to the time commitment issue. Yet some participants called them “boring” (Participant 3, female, 65), “schedule restricting” (Participant 24, female, 77), “too detailed” (Participant 25, female, 70), and leading to a memory bias (Participant 4, male, 83).

## Discussion

The older adults who were part of this study used a wide spectrum of traditional media and ICTs and multitasked with them. Although research with bigger samples of older adults with different socioeconomic and cultural backgrounds should be conducted in the future to support this conclusion, we suggest that it is too early to discard older media/ICT forms when studying multitasking. Whereas contemporary media-use research is focused on older adults’ adoption of newer ICTs, traditional media/technologies, such as print, radio, music, and television, remains widely used sources of information and enjoyment for participants in this study. Given that new ICT use in older adults is positively associated with higher education, higher income, good health, and belonging to their society’s racial or ethnic majority (Elliot, Mooney, Douthit, & Lynch, 2014), all of which are characteristics of our sample, we suggest that traditional media may play a more important role in lives of older adults from other socioeconomic and cultural backgrounds. Future studies must explore how older adults with diverse levels of education and income as well as who represent minority racial/ethnic and disability groups engage in and make sense of media/ICT multitasking.

Our participants showed a great loyalty to print media. They tended to read newspapers, magazines, and books in a low-distraction setting that allowed them to focus on printed content. Print media use, even when combined with other activities, was often perceived as the primary task. This finding has an important practical implication with regard to communicating mediated messages to older individuals similar to those in our sample. To reach this demographic group, it is crucial to continue using newspapers as a key medium to place targeted messages.

Although print media may have a high cognitive value for some older adults, radio, music, and television are used for background noise. This finding mirrors the results of other researchers (Niemelä et al., 2012). The cognitive value of multitasking with these media seems to be small—users do not process information from these sources thoroughly. Yet they enjoy the “noise” because it “helps” them to get through boring tasks, doubles the pleasure of engaging in tasks with which the noise is combined, and decreases loneliness.

One interesting finding of this study supported in previous literature (Voorveld & van der Goot, 2013) was that our interviewees mostly multitasked with familiar traditional media. They preferred that to multitasking with newer ICTs, possibly because older media did not greatly interfere with primary activities and did not pose a significant cognitive burden. Newer ICTs seemed to require attention switching, taking a break rather than engaging in concurrent exposure. For example, participants would take breaks to play solitaire on the computer, check social media and E-mail, and browse the Internet. Traditional electronic media, such as radio and television, always play in the background, ensuring simultaneous exposure. Notably, the love of traditional media content, mostly music, drove our interviewees to purchase newer ICTs, such as MP3 players or satellite radio subscriptions, to make audio content more mobile and omnipresent. This finding has a great practical implication for adoption of newer ICTs among older adults. Focusing on audio content, such as podcasts and audio books, may increase the rates of ICT use among those aged 65 or older. Therefore, to increase new ICT adoption rates, we suggest introducing familiar traditional content through the newer channels.

Our study offers qualitative evidence that media multitasking is an intergenerational behavior. Most of our interviewees, who were from 65 to 90 years old, said that they had been multitasking with media for decades. Many of the participants expressed awareness of potentially negative effects of multitasking and possible cognitive bias that comes with it. Yet they found themselves “doing that anyway” (Participant 28, female, 65). One participant (Participant 4, male, 85) described multitasking as a biological, “animalistic” need. When to multitask and when to stop is decided intuitively, using a sixth sense, and depends on the nature and difficulty of a task and the nature of mediated content (e.g., music) rather than the device/technology itself. These findings echo the results of qualitative research with members of younger generations (college students) that suggests that while younger adults understand the pitfalls of multitasking, they continue to multitask and find positive rewards in doing it (Bardhi et al., 2010).

Some participants explained that the habit to multitask depended on personality, gender, and age. Although numerous studies have explored relationships between these factors and multitasking behavior, these previous studies mostly focused on younger adults and general populations (e.g., Foehr, 2006; Kononova, 2013). Additional research should be done with samples of older adults, particularly, diverse samples of older adults, who may be more likely to use media/ICTs in different ways than did the participants in this study, all of whom were white. This lack of diversity is one of our study’s limitations. For example, media multitasking research with younger populations has shown that socioeconomic status predicts personal ownership of media/ICTs that, in turn, predicts media multitasking (Jeong & Fishbein, 2007; Kononova, 2013). Studying populations that represent lower socioeconomic levels may discover lower levels of new ICT ownership and, thus, fewer opportunities to engage in multitasking. Alternatively, differences may exist in the patterns of multitasking among older adults with different levels of education and income. Representatives of higher socioeconomic status (SES) groups may combine other activities with newspaper and magazine reading, whereas those of lower SES may be more likely to pair radio and television use with other tasks. Several male participants described their female partners to be better at adopting new technologies and combining their use with other activities. This finding calls for further investigation of the role that domestic partners play in new media and technology adoption among older adults.

The findings of the present study dispel the myth that older adults do not multitask with media and ICTs. We suggest that they do, but they mostly engage in concurrent activities using traditional media. The challenge for researchers is in grasping the patterns of these complex multitasking activities. As cognitive ability deteriorates with age and complex media-use behaviors impose an additional cognitive burden that makes recollection of these behaviors less accurate, researchers are challenged with identifying a method of data collection acceptable to participants that does not negatively affect data quality. The first step in this direction is to ask the multitaskers themselves how they think media- and ICT-related multitasking activities should be studied in their age group. Our participants suggested that participant (noncamera) observation and keeping paper-and-pencil diary would be the most acceptable methods to use to study complex media use. They resorted to choosing older, more familiar, methods (some of our participants were familiar with Nielsen’s diary studies, for example). Choosing between less accurate methods of data collection, such as observation and diary keeping, and intrusive data-logging methods, the interviewees clearly preferred the former. The fear of compromising privacy was greater than the enthusiasm for “objective” science. Additional obstacles mentioned were fatigue of the participants (diary), space limitations (observation), and time commitment (all methods).

Some ways to overcome these obstacles included (1) showing footage of recorded data to a participant for edits and the opportunity to withdraw before releasing the data to the researcher, (2) paying extra to reimburse for “invasion,” (3) obtaining consent from all members of household about conducting the study, (4) giving more time flexibility in scheduling research sessions with participants, and (5) assuring confidentiality of any collected data.

### Limitations

Limitations of this work include limited sample size and the homogeneity of the sample. The size of the sample is less of a challenge because we reached the point of saturation during the latest in-depth interviews. We, however, acknowledge that saturation could have been reached because the sample was homogeneous. The results are limited to a group of white U.S. participants who are more likely to be affluent and familiar with newer technologies. Additionally, our participants represented a small geographical area, which was only one of many midsized media markets in the United States. The same interviews should be conducted in the future in other media markets to ensure the diversity of findings. Sample homogeneity and constrained geographic area limitations are especially relevant to the findings related to multitasking research methods because expectations for compensation of study participation may differ from one geographical region to the other, based on lifestyle, overall income levels, and cost of living. Finally, although the qualitative nature of this work enabled us to collect multifaceted information from our participants, quantitative studies with large, representative samples are warranted.
